# Radiomics of T2-weighted MRI for pretreatment prediction of prognosis and temozolomide chemosensitivity in glioma

**DOI:** 10.1186/s12885-026-15726-8

**Published:** 2026-02-17

**Authors:** Chongshun Zhao, Ruoyu Huang, Xiaopeng Li, Ke Tang, Jinli Ding, Yiming Li, Zhongliang Cui, Zheng Zhao, Wei Zhang, Zenghui Qian

**Affiliations:** 1 Department of Neurosurgery, Beijing Tiantan Hospital, Capital Medical University, No. 119 South Fourth Ring Road West, Fengtai District, Beijing, PR China; 2 Department of Neurosurgery, Chinese PLA General Hospital, 28 Fuxing Road, Beijing, PR China; 3 Department of Radiology, Beijing Tiantan Hospital, Capital Medical University, Beijing, China; 4 Beijing Neurosurgical Institute, Capital Medical University, Beijing, 100070 China

**Keywords:** Glioma, Radiomics, MRI, Prognosis, Chemosensitivity

## Abstract

**Background:**

We aimed to construct and validate a radiomics prediction model based on preoperative T2-weighted MRI for prognosis and chemosensitivity prediction in patients with glioma.

**Methods:**

A total of 576 glioma patients were enrolled in this study. The training and validation group included 324 patients and 127 patients respectively with preoperative MRI image data, tumor transcriptome sequencing data and clinical information. The prospective validation group consisted of 125 patients with preoperative MRI image data and clinical information. The radiomics prediction model was constructed based on the prognostic relevant radiomic features of glioma patients in the training group. The radiomics prediction model was validated inpatients of retrospective and prospective validation groups. Functional annotation of radiomic features was performed by pearson correlation analysis of biological process scores and radiomic features values of patients in the training group and validated by transcriptome sequencing, single cell sequencing, reactive oxygen species detection and endoplasmic reticulum stress detection of tumors of patients in retrospective and prospective validation groups.

**Results:**

The radiomics prediction model, which consisted of 17 radiomic features, showed highly predictive stability in overall survival and progression-free survival prediction. Compared with patients who underwent postoperative radiotherapy alone, only patients in the high-risk group benefited from postoperative chemoradiotherapy. The prognostic relevant radiomic features were closely related to immune response, reactive oxygen species metabolism and endoplasmic reticulum stress of glioma cells in silico and in vitro.

**Conclusions:**

The radiomics prediction model serves as a non-invasive tool to predict prognosis and temozolomide chemosensitivity of glioma patients based on preoperative T2-weighted MRI.

**Supplementary Information:**

The online version contains supplementary material available at 10.1186/s12885-026-15726-8.

## Background

Glioma is the most common entity of the tumors within the central nervous system (CNS) [1]. After the maximal safe resection of the tumor, postoperative adjuvant radiotherapy and/or chemotherapy is the standard treatment for glioma [2]. Given the variable prognosis and chemosensitivity in different patients, evaluation of the patient’s response to chemotherapy before postoperative treatment is quite necessary. Although some molecular pathological findings, such as MGMT promoter methylation and IDH1 mutation status, have been found to be predictors of prognosis and chemosensitivity, accurate detection of these molecular pathological markers required professional technical staffs, expensive devices and costly kits [3, 4]. These shortcomings limit the widespread use of molecular pathological markers in predicting prognosis and chemotherapy sensitivity.

Radiomics, which involves the extraction of quantitative features from radiographic medical images through the application of data-characterization algorithms, serves as a pivotal technique for the development of prognostic predictive models and aids in the formulation of therapeutic decision-making strategies within the oncology field [5–7]. The original state of tumor is well reflected by preoperative radiomic features, which allows evaluation of the tumor biologic characteristics and its microenvironment [8, 9]. Magnetic Resonance Imaging (MRI) is highly regarded for its diagnostic accuracy in identifying glioma, and it is commonly employed to ascertain the tumor’s precise location and extent. Recent studies have demonstrated that MRI-based radiomic features may hold significant potential as indicators for both prognostic and predictive biomarker assessment in glioma [10, 11]. Although some prognostic or biomarkers prediction models for glioma have been developed, to the best of our knowledge, there is currently no model for predicting the sensitivity of gliomas to temozolomide chemotherapy, and there is not yet a radiomics prognostic or treatment guidance model that is powerful enough to be applied in clinical practice.

Therefore, the aim of this study was to construct and validate a prediction model based on preoperative T2-weighted MRI image from glioma patients that incorporated prognosis and chemosensitivity prediction in glioma. The stability of the predictive model was validated in independent and prospective validation groups. Methods such as single-cell sequencing and laboratory tests are used to provide biological interpretations of the radiomic features included in the prognostic model. In summary, a radiomics prediction model was built to change the clinical management of glioma.

## Patients and methods

### Patients and tumor sequencing

This study involved three independent patient cohorts: a training cohort, a retrospective validation cohort, and a prospective validation cohort. The training cohort was established from two public databases. The training cohort consisted of 60 glioma patients from the CGGA325 dataset and 264 glioma patients with T2-weighted MRI data from the TCGA database, among whom 189 patients also had tumor transcriptome sequencing data. The retrospective validation cohort consisted of 127 glioma patients from the CGGA693 dataset, all of whom had complete T2-weighted MRI and tumor transcriptome sequencing data. The median follow-up time for the entire retrospective cohort (training and retrospective validation groups) was 37.4 months (95% CI: 32.8–42.0 months). For the prospective validation cohort, 314 consecutive glioma patients were enrolled at Beijing Tiantan Hospital from November 2016 to April 2018. After applying the exclusion criteria, 125 patients were ultimately included. Follow-up for this prospective cohort was conducted trimonthly via telephone or clinic visits, with a median follow-up of 48.3 months (95% CI: 47.4–51.5 months). A detailed flowchart of patient inclusion and exclusion is provided in Supplementary Fig. 1. The demographic and clinicopathologic characteristics of all patients are summarized in Supplementary Table 1.

In validation group, tumor samples obtained from surgery were swiftly immersed in liquid nitrogen for preservation. Transcriptome data of patients in validation group were generated using the Illumina sequencing platform. Two expert neuropathologists were responsible for rendering the pathological diagnoses of the tumor samples. The molecular pathology examinations were carried out at the Molecular Pathology Testing Center affiliated with the Beijing Neurosurgical Institute.

In prospective group, the acquisition of multi-site samples was carefully designed before surgery and was completed under the guidance of intraoperative neuronavigation. Single cell sequencing was performed on Illumina platform. Definition of single cell types was performed by t-SNE analysis.

### Radiomic features extraction

The tumor region of interest (ROI) was delineated on T2-weighted MR images, as this sequence is widely accepted for identifying glioma regions. ROIs were manually delineated by two experienced neuroradiologists (both with over 10 years of practice), using the MRIcron software (http://www.mccauslandcenter.sc.edu/mricro). Raters were instructed to delineate the entire visible hyperintense tumor region on T2-weighted images, excluding surrounding edema, cerebrospinal fluid, and large cystic or necrotic cavities. For the training cohort derived from public databases (TCGA and CGGA), images were acquired from multiple institutions using various MRI scanners with field strengths of 1.5T or 3.0T. For the prospective validation cohort enrolled at Beijing Tiantan Hospital, MRI acquisition was performed on a 3.0 T MR scanner (Ingenia CX, Philips Healthcare, Best, the Netherlands) with a 32-channel head receiver coil. Radiomic features were extracted from the axial 2D Turbo Spin Echo (TSE) T2-weighted images. The specific acquisition parameters were: repetition time (TR) = 2800 ms, echo time (TE) = 135 ms, flip angle = 90°, field of view (FOV) = 240 × 240 mm², matrix size = 240 × 240, and slice thickness = 5 mm with an inter-slice gap of 0.5 mm (acquired voxel size = 1 × 1 × 5 mm³).

To address the heterogeneity of image acquisition parameters across cohorts and adhere to the Image Biomarker Standardisation Initiative (IBSI) guidelines, a standardized preprocessing pipeline was implemented prior to feature extraction. First, N4 bias field correction was applied to correct magnetic field inhomogeneity. Second, all images were resampled to a uniform voxel size of 3 × 3 × 3 mm**³** using B-spline interpolation to standardize spatial resolution. Third, to normalize intensity scales across different scanners, image intensities were normalized using Z-score normalization (zero mean and unit variance) based on the intensity values within the brain mask.

For each patient, 1733 radiomics features were extracted using the “PyRadiomics” package (version 2.2.0) in Python [12]. The extracted features were categorized into four groups: (1) First-order statistics: *n* = 18; (2) features related to shape and size: *n* = 13; (3) Textural features, which were derived from texture matrices including gray-level co-occurrence matrix (GLCM), gray-level run length matrix (GLRLM), gray-level size zone matrix (GLSZM), gray-level dependence matrix (GLDM): *n* = 68; (4) Features derived from different filters, including: filter “wavelet”: *n* = 688; filter “LBP”: *n* = 258; filter “LoG”: *n* = 258; other filter (“square”, “squareroot”, “logarithm”, “exponential”, “gradient”): *n*=86 × 5 = 430. The detailed setting of feature extraction was provided in the Supplementary Material, and the detailed calculation formula for each radiomic feature was provided on the official website (https://pyradiomics.readthedocs.io).

### Prediction model construction

The risk prediction model was developed utilizing radiomic features (RFs). To ensure the robustness of the model, the RFs underwent a rigorous two-stage selection process within the training group. Firstly, in order to reduce the influence of variations between neuroradiologists, we calculated the intraclass correlation coefficient (ICC) to assess the stability of each feature and only those with high stability (ICC > 0.9) entered following processes of feature selection and model establishment. The second step was to screen out RFs associated with poor prognosis in both CGGA and TCGA databases. Finally, 17 RFs were screened out for the risk prediction model construction. To accurately weight the independent contribution of each feature, a multivariate Cox proportional hazards regression analysis was performed using these 17 features in the training cohort. The risk score for each patient was calculated as a linear combination of the feature values weighted by their corresponding multivariate regression coefficients ($\:\beta\:$). The formula is as follows: $\:\mathrm{Risk\:Score}=\:\sum\:\left({\beta\:}_{i}\times\:{\mathrm{Feature}}_{i}\right)$. Patients were stratified into high-risk and low-risk groups based on the median risk score of the training cohort. This cutoff method was selected to prevent data dredging and ensure the robustness of the model in validation cohorts.

### Functional annotation of radiomic features

Functional annotation of the radiomic features was performed through Gene Set Variation Analysis (GSVA) coupled with *pearson* correlation analysis. Initially, the activation scores for biological processes and pathways for each patient were determined by GSVA, utilizing data from tumor transcriptome sequencing. The gene sets of Gene ontology (GO) and Kyoto Encyclopedia of Genes and Genomes (KEGG) were downloaded from Gene Set Enrichment Analysis (GSEA) Web portals (http://software.broadinstitute.org/gsea/index.jsp). Subsequently, the correlation between these activation scores and the radiomic feature values was assessed using Pearson correlation analysis. The functions and pathways that demonstrated significant correlation with the radiomic features were then employed for their annotation. Classification of these biological functions was performed according to the classification in the AmiGO 2 portal (http://amigo.geneontology.org/amigo).

### Glioma cell culture

Patient-derived glioma cells (PDCs) was also established according to the previous study [13]. PDC were cultured in serum-free medium containing DMEM/F12 (Gibco) supplemented with B27 (Gibco), basic fibroblast growth factor (bFGF, 20 ng/mL), epidermal growth factor (EGF, 20 ng/mL) and heparin (2.5 mg/mL). Growth factors (bFGF and EGF) were added twice a week. PDCs were enzymatically dissociated into single cells using Accutase (Sigma Aldrich) and thereafter routinely cultured in the serum-free medium every 4–6 days. All PDCs could stably grow and passage were selected for functional verification.

### Reactive oxygen species detection

Reactive oxygen species of PDCs was detected by reactive oxygen species assay kit (C1300, applygen). The experimental process was carried out strictly accordance with the instructions. DCFH-DA was added to the cell suspension to the final concentration 10 µM. Immediately after the addition of the reaction reagent, the cells were incubated at 37 °C for 60 min. The result was detected by multiscan spectrum (M200 Pro, Tecan). The excitation wavelength is set to 500 nm and the emission wavelength is set to 525 nm.

### Endoplasmic reticulum stress detection

Reactive oxygen species of tumor cells was detected by western blot. The protein extracted from Patient-derived glioma cells. Primary and secondary antibodies were from Endoplasmic reticulum (ER) Protein Folding Antibody Sampler Kit (4759T, Cell Signaling Technology). The result was detected by motored molecular imaging system (Bio-Rad Laboratories).

### Protein modification validation

The correlation between protein expression values and RF values were calculated by *pearson* correlation analysis. A matrix of correlation coefficients was obtained. Subsequently, unsupervised cluster analysis based on the coefficient matrix was performed. The protein expression data was obtained from TCGA database.

### Statistical analysis

Statistical analyses and data visualization were performed by software environment R (v3.5.0), SPSS software (v25.0, IBM) and Office 2016 (Microsoft). Student’s t test was used to validate differences between two variables. Chi-square test was used to assess the composition ratio differences between two groups. The log-rank test was used to assess the statistical significance between survival groups in Kaplan-Meier survival analysis. Internal validation was performed using bootstrap resampling (1,000 iterations) to calculate the optimism-corrected C-index. Multivariate Cox regression analysis was used to determine the coefficients of the radiomic features for the risk score construction. The primary endpoints were overall survival (OS) and progression-free survival (PFS). OS was defined as the time from the date of surgery to the date of death from any cause. PFS was defined as the time from the date of surgery to the date of disease progression or death. Patients who had not experienced an event at the time of the final analysis were censored at the date of their last follow-up. A *p*-value of less than 0.05 was set as the threshold for statistical significance. All statistical analyses were reviewed by a statistician.

### Prospective study design

A single-center prospective study was performed to assess the clinical usefulness of the radiomics prediction model. This study was designed as an observational study. The treatment strategies for all patients in the prospective cohort were determined by a multidisciplinary team based strictly on clinical guidelines and the standard of care at our institution. The radiomics risk scores were calculated post-hoc using the same parameters and cutoff values in the training group, and were not available to the treating physicians or patients at the time of treatment decision-making. Therefore, the risk scores did not influence the clinical management.

## Results

### Constructing a risk prediction model based on radiomic features

**Fig. 1:**
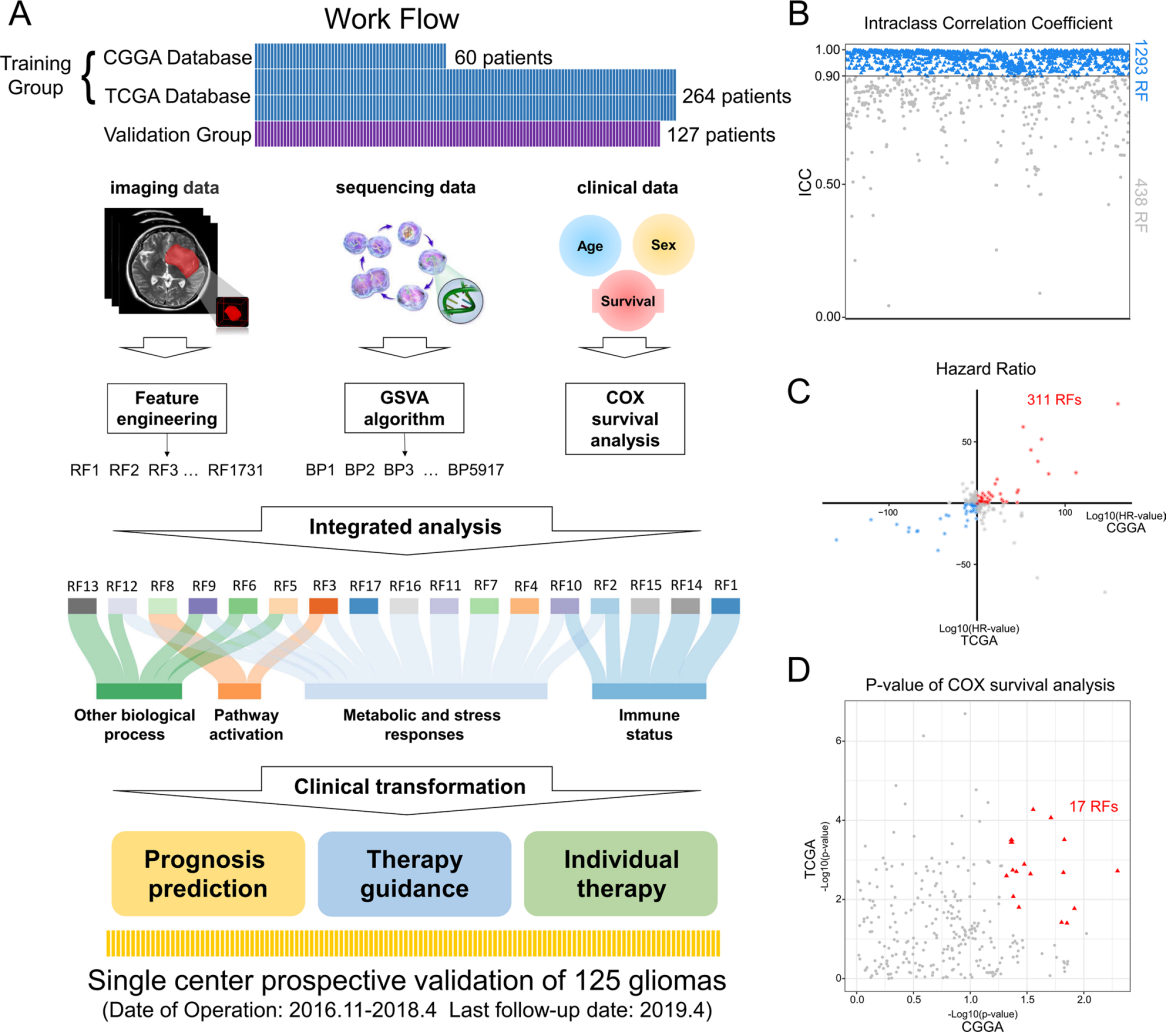
Work flow and radiomic feature screening. **A** Work flow of this study. There were three groups of patients in this study, training group, validation group and prospective group. Highly stable and prognostic-related 17 RFs were screened out based on training group: In training group, predictive models based on RFs were built for prognosis prediction, therapy guidance and individual therapy. Prediction models were verified in retrospective and prospective validation groups. Finally, biological annotation of 17 RFs was performed and validated. **B** 1293 RFs showed high intraclass correlation coefficient between tumors sketched by radiologist or neurosurgeon. **C** 311 RFs associated with poor prognosis in training group. **D** 17 RFs were significantly associated with poor prognosis steady in training group

The workflow of this study was shown in Fig. 1. First step, 1731 radiomic features (RFs) and 5917 biological process scores (BPs) of each patient were calculated. The algorithm was detailed in the method section. Second step, the tumors in T2-weighted MRI images were sketched by a radiologist and a neurosurgeon respectively. Only 1293 RFs with high intraclass correlation coefficient (>0.9) were selected for subsequent analysis (Fig. 1B). Third step, 17 RFs, as poor prognosis factors by univariate COX analysis in both CGGA and TCGA databases in training group, were screen out (Fig. 1C and D). The stability of these 17 RFs as predictors of poor prognosis was confirmed in the validation group. The results of COX analysis were shown in Supplementary Table 2. Based on these 17 RFs, a risk prediction model was constructed. Fourth step, verifying the performance of the risk prediction model in training and validation groups and applying the risk prediction model to clinical practice. Finally, biological annotating of the 17 RFs was further validated by single-cell RNA-sequencing data and patient-derived cells (PDCs).

### Performance of the risk prediction model in prognosis prediction and treatment guidance in retrospective analysis

**Fig. 2:**
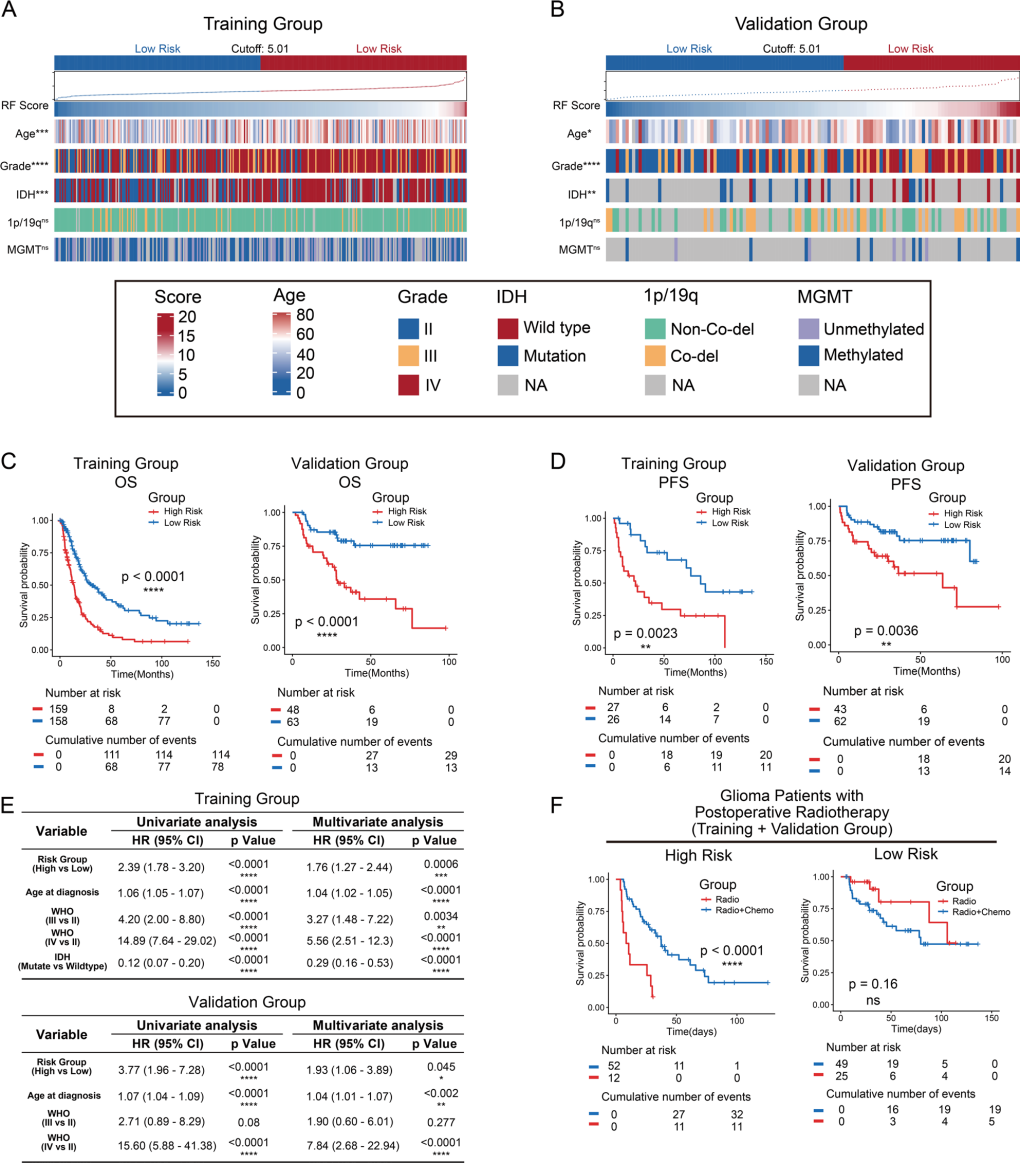
Clinical pathological and survival differences in different risk groups. **A**, **B** The differences of survival and clinical molecular pathology in patients in different risk groups were showed in heatmaps. Patients in training and validation groups were arranged in ascending order of risk scores of RFs. **C** Kaplan-Meier curves showed the overall survival of patients in low risk and high risk groups. Overall survival of patients in high risk group was significantly shorter. **D** Kaplan-Meier curves showed the overall survival of patients in low risk and high risk groups. Progression-free survival of patients in high risk group was significantly shorter. **E** Univariate and multivariate COX survival analysis of the predicted risk group and other prognostic factors in both training and validation groups. **F** Kaplan-Meier curves showed the overall survival of patients in high risk group received chemoradiotherapy was significantly longer than patients received radiotherapy. Patients in low risk group did not show significant differences in the benefits of the two treatments. *: *p* < 0.05, **: *p* < 0.01, ***: *p* < 0.001, ****: *p* < 0.0001, ns: no significant difference

The correlation between the risk scores generated by the predictive model and the clinicopathological characteristics of the patients was depicted in the heatmaps (Fig. 2A and B). patients were arranged in order of increasing risk scores. In terms of model performance, the C-index of the model in the training group was 0.627 (95% CI: 0.587–0.669), and 0.710 (95% CI: 0.623–0.796) in the validation group. Time-dependent ROC analysis demonstrated stable predictive power, with 1- and 3-year AUCs of 0.66 and 0.71 in the training group, and 0.68 and 0.73 in the retrospective validation group. (Supplementary Fig. 2A, B). Furthermore, the calibration plots showed good agreement between the predicted and observed survival probabilities (Supplementary Fig. 2C, D). Decision Curve Analysis (DCA) indicated that the radiomics model provided a higher net benefit than the treat-all or treat-none strategies across a wide range of threshold probabilities, supporting its clinical utility (Supplementary Fig. 2E, F). The median risk score (5.01) from the training group was established as the threshold for stratifying patients into different risk groups. Patients with risk scores above 5.01were allocated to the high-risk group, while those scoring below 5.01 were placed in the low-risk group. The distribution of age, Grade, IDH mutation status was uneven across the two risk groups. In contrast, there was no statistically significant difference in the 1p/19q status and MGMT between the two groups. Subsequently, prognostic analysis in training and validation groups showed that patients in high risk group had shorter overall survival and progression-free survival than those in the low risk group (Fig. 2C and D). Univariate and multivariate COX survival analysis showed that risk group was an independent prognostic factor after adjusted other prognostic factors in both training and validation groups (Fig. 2E). Importantly, we found that patients in different risk group exhibited varying benefits from postoperative temozolomide treatment. Specifically, among patients received the same radiation regimen after surgery, only those classified as high risk could benefit from postoperative temozolomide treatment (Fig. 2F).

### Performance of the risk prediction model in prognosis prediction and treatment guidance in prospective analysis

**Fig. 3:**
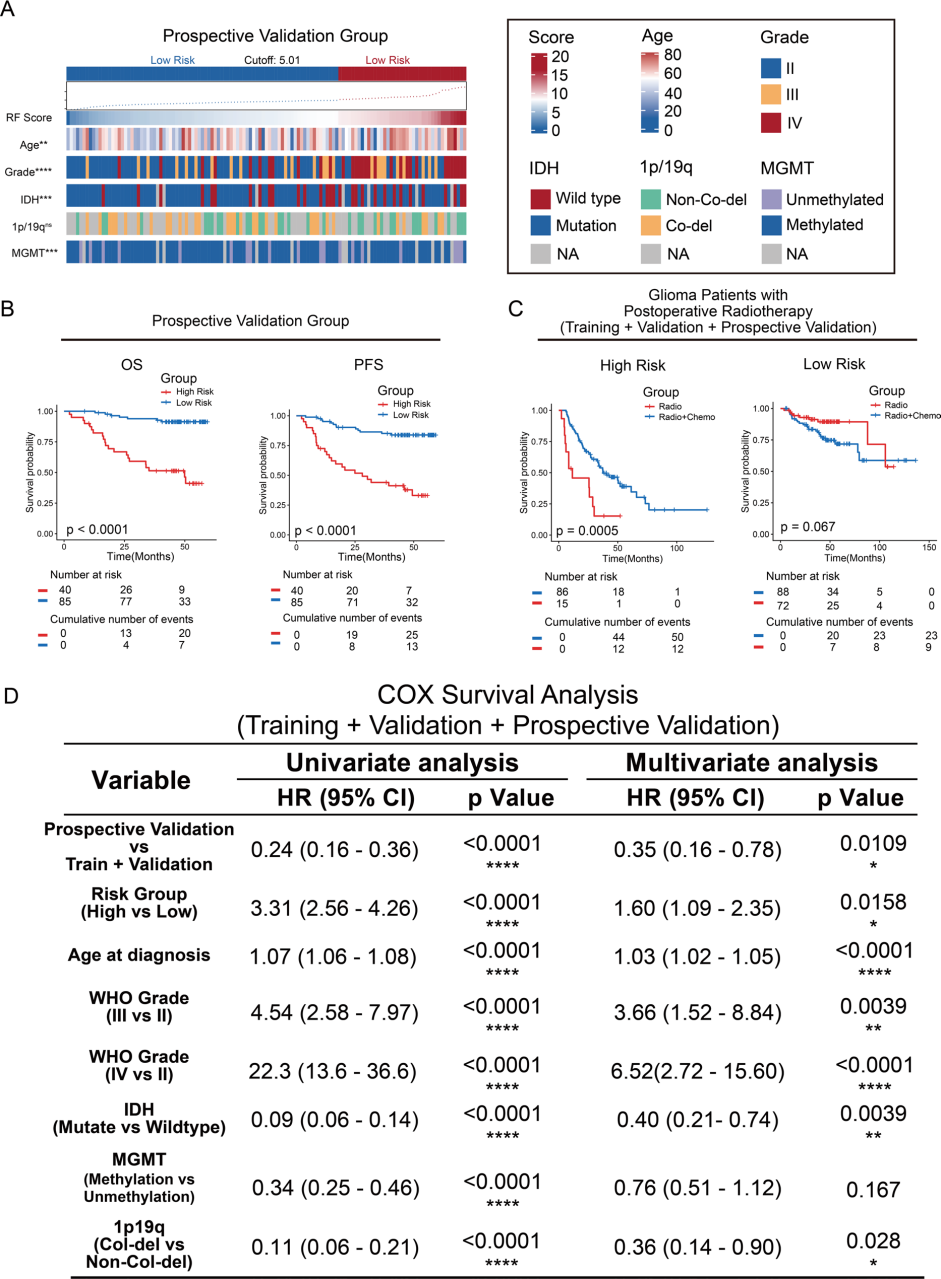
The stability of the predictive models was verified in prospective group. **A** The differences of survival and clinical molecular pathology in patients in different risk groups were showed in heatmaps. Patients in training and validation groups were arranged in ascending order of risk scores of RFs. **B** Kaplan-Meier curves showed the overall survival and progression-free survival of patients in high risk group was significantly shorter than those in low risk group in prospective group. **C** Kaplan-Meier curves showed the overall survival of patients in high risk group received chemoradiotherapy was significantly longer than patients received radiotherapy in validation and prospective group patients. **D** Univariate and multivariate COX survival analysis showed that treatment stratification in whole cohort was independent prognostic factor. *: *p* < 0.05, **: *p* < 0.01, ***: *p* < 0.001, ****: *p* < 0.0001, ns: no significant difference

To further validate the stability and reproducibility of risk prediction model, a single-center prospective analysis was performed. The correlation between the risk scores generated by the predictive model and the clinicopathological characteristics of the patients was depicted in the heatmaps (Fig. 3A). The model exhibited superior performance in the prospective validation cohort, achieving a C-index of 0.798 (95% CI: 0.718–0.871) and AUCs of 0.87 and 0.85 for 1- and 3-year survival, respectively (Supplementary Fig. 2A, B). Consistent with the retrospective cohorts, the prospective cohort also demonstrated excellent calibration and significant clinical net benefit in DCA (Supplementary Fig. 2C-F). Prognostic analysis showed that patients in the high risk group had shorter overall survival and progression-free survival (Fig. 3B). As the number of patients in prospective group was too small to allow survival analysis, those patients were integrated with patients in retrospective groups for the analysis of chemosensitivity. The comparison of clinicopathological characteristics between patients who received chemotherapy and those who did not in different risk groups is summarized in Supplementary Table 3. After increasing the sample size, we analyzed patients who underwent postoperative radiotherapy. By comparing overall survival between those receiving radiotherapy alone and those receiving concurrent/adjuvant chemoradiotherapy (Radiotherapy + TMZ) within the same risk strata, we determined that only the high-risk group derived significant survival benefit from the addition of temozolomide (Fig. 3C). To clarify whether the clinical application of this predictive model could benefit glioma patients, the differences in the patient’s postoperative treatment were analyzed. Although this is an observational study and the treatment decisions were made independently of the radiomics model, postoperative treatment of patients in the prospective group were more likely to the recommended treatment plan of the risk prediction model (Supplementary Table 1). A higher proportion of patients, who have undergone surgery after Nov. 2016 and classified into low risk group received postoperative radiotherapy alone, while patients in the high risk group received radiochemotherapy. As shown in Fig. 3D, after adjusting for key clinicopathological covariates including Age, WHO Grade, IDH mutation status, MGMT promoter methylation, 1p/19q co-deletion, and Cohort source, the RF Group remained a statistically significant independent prognostic factor (HR = 3.31, 95%CI 2.56–4.26, *P* < 0.0001). This indicates that the prognostic value of the radiomics model is robust and not merely a surrogate for other molecular or clinical features.

### Overview of the prognostic radiomic features related biological processes

**Fig. 4:**
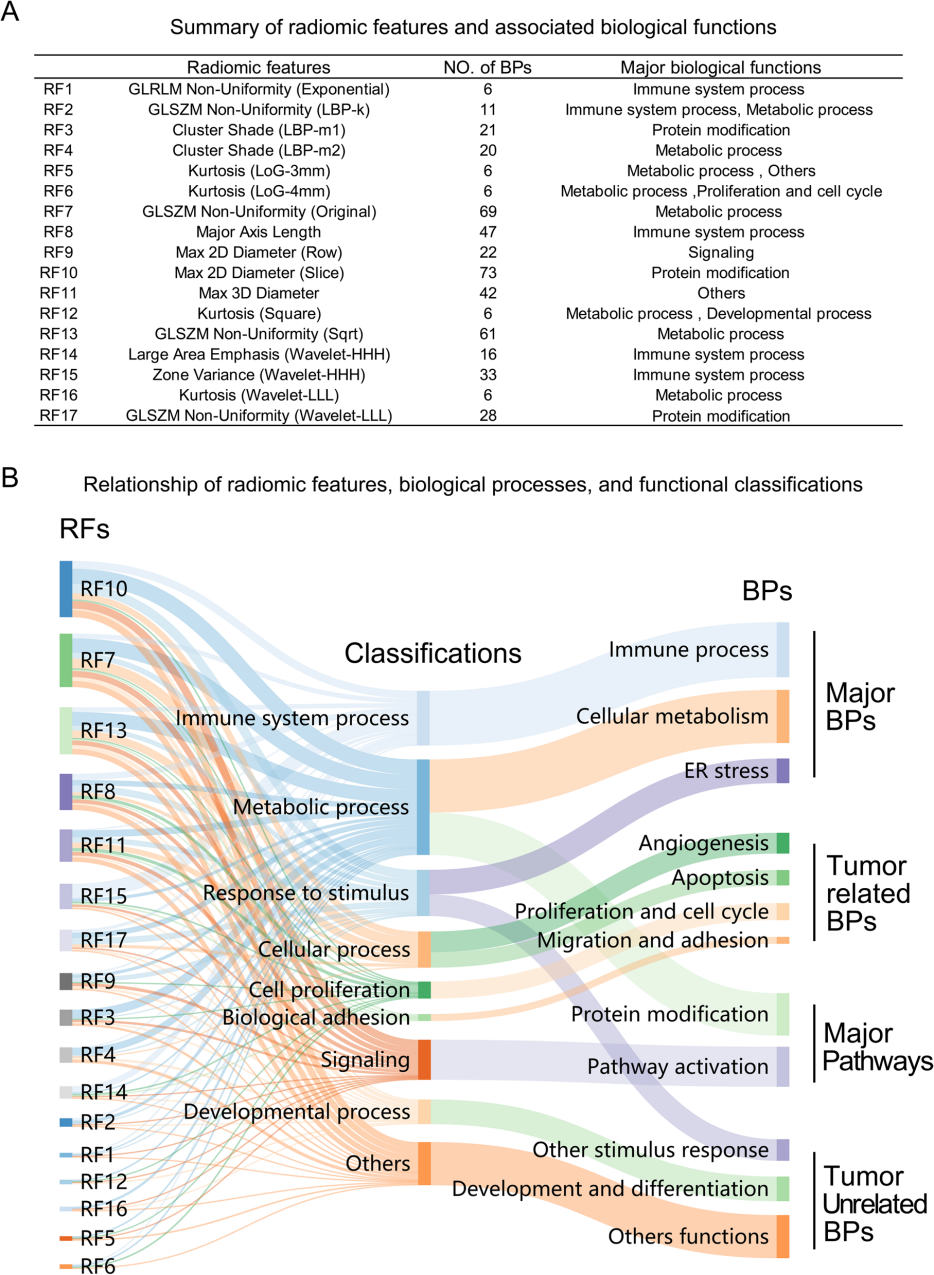
The relationship between RFs and biological processes of tumor. **A** 17 RFs of risk predict model and their corresponding radiomic feature names, numbers of associated BPs and major biological functions. Major biological functions were the classifications of biological processes most associated with the corresponding RFs. **B** Sankey Diagram showed the classifications of RFs related BPs. The RFs related BPs colud be divided into four categories. BPs: biological processes; ER: endoplasmic reticulum; GLSZM: Gray Level Size Zone Matrix; GLRLM: Gray Level Run Length Matrix; GLCM: Gray Level Co-occurrence Matrix; LBP: Local Binary Pattern; LoG: Laplacian of Gaussian; Sqrt: Square Root; HHH/LLL: Wavelet decompositions (High/Low frequency sub-bands)

In order to functionally annotation of RFs in glioma, the correlation between RFs and molecular pathology and biological processes were analyzed. Surprisingly, almost all RFs showed no correlation with WHO grade, IDH mutation status and 1p/19q status in both training group and validation group. The results were shown in Supplementary Table 4. To evaluate the biological basis of the prognostic radiomic features, 17 RFs of 189 patients in training group and 127 patients in validation group were analyzed with matched 5917 BPs. Although 17RFs were the most relevant factor for poor prognosis of glioma, they showed great differences in biological annotation analysis (Fig. 4A). On the one hand, the number of BPs associated with different RF varies widely, from 6 to 73. Some RFs were significantly associated with many BPs, such as RF10, RF7, and RF13. However, some RFs were only significantly associated with a small number of BPs, such as RF1, RF5, RF6, RF12 and RF16. On the other hand, BPs related to different RFs also varies widely. As shown in Fig. 4B, RFs-related BPs could be classified into nine classifications and four categories. The most relevant BPs to these 17 RFs were named Major BPs (Immune process, cellular metabolism and endoplasmic reticulum stress) and Major Pathways (Protein modification and pathway activation). The remaining BPs could be divided into tumor related BPs and tumor unrelated BPs. The following studies focused on RFs related Major BPs, Major Pathways and Tumor related BPs.

### Biological interpretation of the prognostic radiomic features

**Fig. 5:**
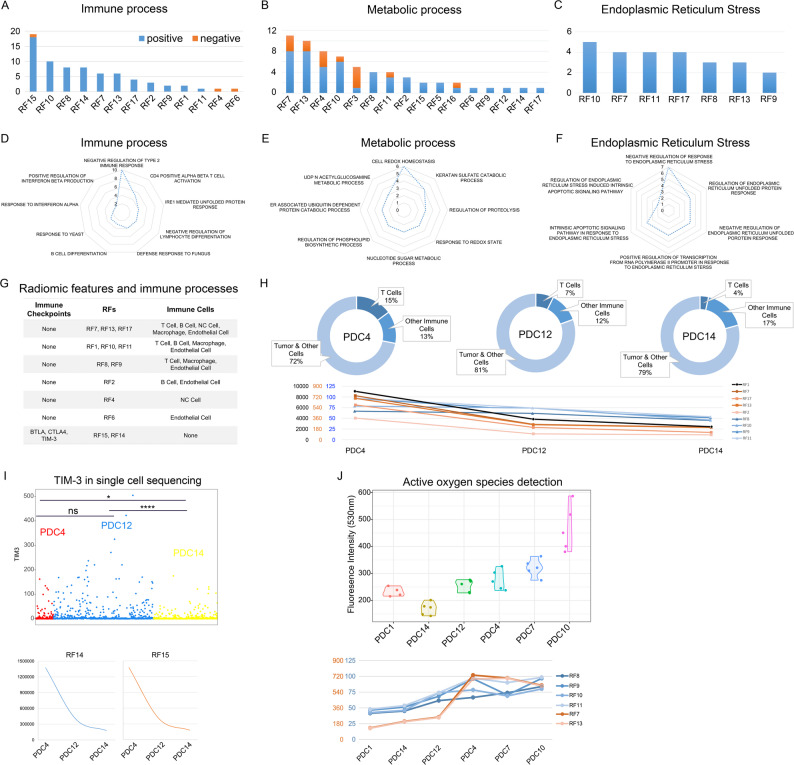
RFs related major BPs and experimental validation. The counts of RFs related BPs in immune process (**A**), metabolic process (**B**) and endoplasmic reticulum stress (**C**). The counts of BPs positively correlated with RFs were marked as blue, and the negative correlated ones were marked as orange. The radar chart showed the counts of BPs most correlated with RFs in immune process (**D**), metabolic process (**E**) and endoplasmic reticulum stress (**F**). **G** RFs that were significantly associated with the expression of immune checkpoints or Immune cell markers. **H** Proportion of cells types, obtained by tSNE analysis based on single cell sequencing data, was showed in the top half of figure. RF values associated with T cell proportion was showed in the lower half of figure. **I** The expression of TIM-3 in single cells of different patients was showed in the top half of figure. RF values associated with the expression of TIM-3 were showed in the figure was showed in the lower half of figure. **J** Fluorescence intensity reflected the ROS of tumors of different patients were showed in the top half of figure. RF values associated with the ROS of tumors were showed in the figure was showed in the lower half of figure

RFs related biological functions were explored in more depth. First, the number of BPs associated with each RF in each main category were showed. As depicted in the Fig. 5A, the most relevant RFs for immune process were RF15 and RF10. The most relevant RFs for metabolic process were RF7 and RF13 and RF10 for endoplasmic reticulum stress (Fig. 5B and C). Second, the most RFs relevant BPs in each main category were also showed. The most relevant immune function of RFs was negative regulation of type 2 immune response (Fig. 5D). Cell redox homeostasis was the most related BPs in metabolic process and negative regulation response to endoplasmic reticulum stress in endoplasmic reticulum stress (Fig. 5E and F). In addition, most RFs relevant BPs in Tumor related BPs were showed in Supplementary Fig. 3. The relationship between RFs and immunosuppression made us think of the role of immune checkpoints in tumor immune microenvironment. Correlation analysis found that only RF14 and RF15 were significantly associated with the expression of immune checkpoints in glioma, while other RFs were associated with the numbers of T cell (Fig. 5G).

### Verification of the relationship between radiomic features and their predicted biological processes

To further validate the biological annotating of 17 RFs, single-cell RNA-sequencing of tumor and PDCs were collected from representative patients in prospective group. Clinical and radiomic features of those patients were shown in Supplementary Table 5. To avoid the influence of tumor heterogeneity on the accuracy of validation results, multi-site single-cell sequencing on tumors of each patient was performed. We found that the biological functions related to RFs showed not differ at different detection sites (Supplementary Fig. 4A, B and C). In Single-cell RNA-sequencing data, the tumor components of the three patients were analyzed and the T cell ratios showed the same trend as the corresponding RFs (Fig. 5H). The expression of TIM-3 in these patients was also analyzed, and the correlation between RF14 and RF15 and TIM-3 expression was verified (Fig. 5I). In patient-derived glioma cells, reactive oxygen species of tumor cells was detected by detection kit. We found that the reactive oxygen species of tumor cells were consistent with the values of the RFs (Fig. 5J).

### Correlation between radiomic features and molecular pathways

**Fig. 6:**
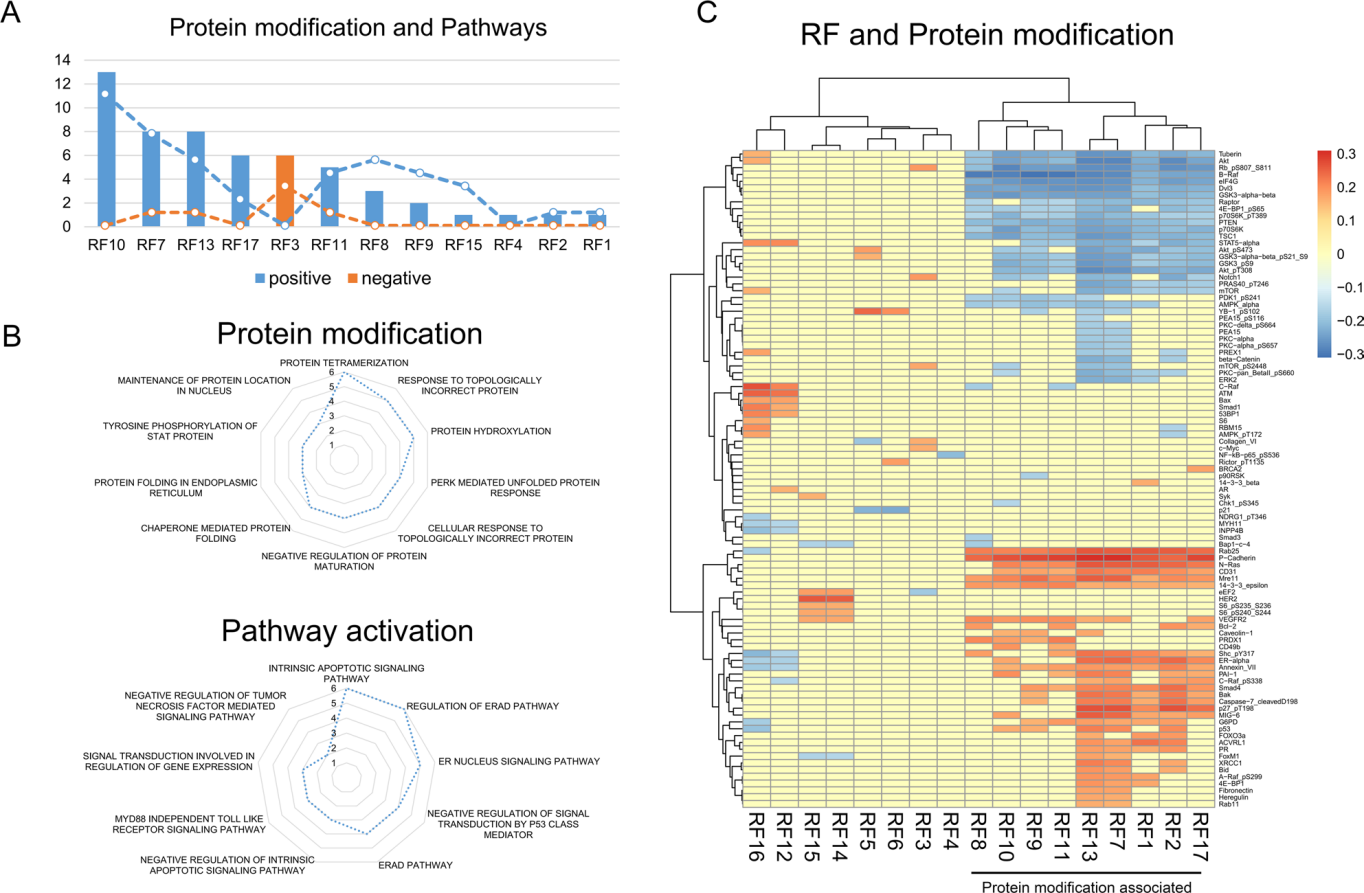
RFs related pathways and protein modification. **A** The counts of RFs related BPs in protein modification (dotted lines) and pathways (columns). The counts of BPs positively correlated with RFs were marked as blue, and the negative correlated ones were marked as orange. **B** The radar chart showed the counts of BPs most correlated with RFs in protein modification or pathway activation. **C** The heatmap showed the result of unsupervised clustering analysis of correlation coefficient between RFs and protein expression

Protein modification is closely related to pathway activation in cancers. This phenomenon could be observed in the RFs correlation analysis. The number of RFs-related protein modifications was highly consistent with the number of RFs-related pathway activations (Fig. 6A). RFs related protein modifications and pathway activations were shown in Fig. 6B. To verify the correlation between RFs and protein modifications, reverse phase protein arrays (RPPA) data was analyzed. Unsupervised clustering analysis of correlation coefficients between RFs and protein expression values of RPPA showed RF10, RF7, RF13, RF17, RF11, RF8, RF9, RF2 and RF1 were classified as protein modification associated RFs, which was almost the same as the above results (Fig. 6C).

## Discussion

MRI is a crucial clinical tool for glioma patients. Preoperative MRI plays a pivotal role in both the diagnosis of gliomas and in guiding tumor resection during surgery through intraoperative neuronavigation. With advancements in radiomics, some studies have found that MRI can be used to predict the genomic and biological features of tumors, such as therapeutic response [14], tumor local recurrent [15], p53 mutation [16] and so on [17–19]. However, radiomic prediction model for postoperative treatment of glioma is still lacking at present. In this study, we developed and validated a prediction model using preoperative T2-weighted MRI of glioma patients for prognosis prediction and therapy guidance. Importantly, this prediction model is stable and easy enough for clinical application.

In our prior research and that of other scholars, multiple radiomic-based prognostic models have been formulated for glioma [20–23]. Our analysis builds on these studies in that we performed a rigorous screening of radiomic features and a comprehensive validation of the predictive model. We developed a robust predictive model utilizing stable predictors, eliminating those with variable performance through a two-step process. The first step excluded the impact of inter-observer variability among neuroradiologists on the stability of RFs. The second step ensured the stability of the predictive model in different populations and different image sources. During the model validation phase, we not only set up an independent validation group but also designed a prospective validation group. In addition, tumor cells from prospective patients were collected for in vitro culture and single-cell sequencing, providing multi-dimensional verification of the stability of our prediction model. In addition to prognosis prediction, we found that the prediction model can also predict the chemosensitivity of patients to temozolomide, the first-line chemotherapy drug for glioma. Patients in the high-risk group were more sensitive to chemotherapy, while the low-risk group patients could not benefit from postoperative chemotherapy. The difference in response to chemotherapy was thought to cause by the difference in the distribution of patients in the high-risk and low-risk groups. However, we found that predicted chemosensitivity was not associated with the grade of tumor in retrospective and prospective analyses. Therefore, we speculated that RFs may be related to the biological behaviors of tumors to predict chemotherapy sensitivity of patients. To test this hypothesis, a functional annotation analysis of the 17 prognostic radiomic features (RFs) was performed.

Mathematically, the 17 prognostic features can be categorized into shape, first-order, and texture features. Shape features (e.g., Maximum 3D Diameter) quantify the geometric extent of the tumor, serving as surrogates for tumor burden. First-order statistics, specifically Kurtosis, measure the ‘tailedness’ of the voxel intensity distribution. High kurtosis indicates a distribution with extreme outliers, mathematically reflecting the presence of hyper- or hypo-intense regions such as necrosis or hemorrhage. Texture features (e.g., Gray Level Non-Uniformity from GLSZM/GLRLM) quantify the variability of gray-level intensity. Higher non-uniformity values mathematically represent a more chaotic arrangement of voxel intensities, which biologically corresponds to high intratumoral heterogeneity. Functional annotation of radiomic features (RFs) remains a formidable challenge within the field of radiomic research. *Roger Sun et al.* have constructed a prediction model to assess tumor-infiltrating CD8 cells and predicts the response to immunotherapy in cancers by integrating analysis of CT images with RNA-seq genomic data [24]. In another study, *Grossmann et al.*. investigated the relationship between RFs and pathway scores derived from gene set enrichment analysis to establish connections between radiomic, pathway, and clinical data [25]. They further substantiated these relationships through immunohistochemical analysis of tumor tissues. Combining the advantages of reported algorithms, functional annotation of RFs was performed based on enrichment scores of 5917 BPs and pathways obtained from RNA-seq data. Consistent with previous reports in other tumors, results showed RFs reflect the tumor immune status, metabolism status, tumor related BPs and pathway activation in glioma [24–26]. In addition, we also found that RFs associated with the endoplasmic reticulum stress state and protein modification status of tumor cells.

To further substantiate these findings, we performed multi-dimensional verification using single-cell sequencing and in vitro functional assays on prospective patient-derived tumors. These analyses confirmed the biological functions inferred from the radiomic features. However, it should be noted that while the RFs effectively reflected the microenvironment and pathway activation, they showed limited performance in predicting the proliferation, migration, and apoptosis status of tumor cells. These results indicate that the prognostic value of the identified RFs in glioma is primarily linked to the tumor microenvironment and protein modification-driven pathway activation, rather than direct measures of tumor growth behaviors.

Previous studies have found that the composition of the tumor microenvironment affects the response of glioma to temozolomide chemotherapy and has a significant impact on the prognosis of glioma patients [27, 28]. However, there is still a lack of indicators to evaluate this phenomenon in clinical practice. These findings suggest that the prognostic value of the radiomics model is partly underpinned by its association with the tumor microenvironment. The observation that low-risk patients did not significantly benefit from adjuvant temozolomide can be explained by both demographic and biological factors. Demographically, the low-risk group was enriched with younger patients and IDH-mutant tumors, which are known to have a more indolent natural history. Biologically, our functional annotation revealed that low-risk tumors exhibit lower levels of ROS metabolism and ER stress. Since alkylating agents like temozolomide work by inducing DNA damage and cellular stress, tumors with a ‘quiet’ metabolic phenotype (low baseline stress) may be less vulnerable to this additional cytotoxic insult compared to the high-stress microenvironment of high-risk tumors. This biological plausibility supports the robustness of the model in predicting patient outcomes and potential response to temozolomide, reinforcing its potential for clinical application. Our prognostic prediction model can accurately predict the prognosis of patients preoperatively and determine whether patients can significantly benefit from postoperative temozolomide chemotherapy, making clinical practice more convenient and effective. Furthermore, compared to the molecular assessment of MGMT promoter methylation, our radiomics-based approach offers distinct advantages. MGMT testing is invasive, subject to sampling errors due to intratumoral heterogeneity, and often limited by the lack of standardized cutoff values and assay variability. In contrast, our model utilizes standard preoperative MRI, providing a non-invasive, whole-tumor assessment that bypasses these technical limitations while accurately predicting the benefit of temozolomide treatment.

Although the prognostic prediction model showed excellent predictive effect in prognosis and chemosensitivity prediction, the current model requires computational software for feature extraction. We are actively developing a mobile application to automate this process. This tool aims to further streamline the workflow, ensuring that the model can be easily integrated into routine clinical practice. Furthermore, our study has limitations regarding tumor taxonomy. Since the training and retrospective validation cohorts were sourced from public databases (TCGA and CGGA) established in earlier eras, complete data for certain molecular markers required for the WHO 2021 classification (e.g., CDKN2A/B homozygous deletion) were not available for all patients. Consequently, we could not strictly reclassify all retrospective cases according to the WHO 2021 standards. Although we adjusted for IDH and MGMT status in our multivariate analyses, this heterogeneity may influence the precise inference of chemotherapy benefit within specific molecular subtypes. However, given that our objective was to construct a generalized prediction model based on preoperative imaging, we believe that this ‘pan-glioma’ approach maintains significant clinical utility in the preoperative setting, where molecular diagnosis is not yet established. Third, although the extent of resection (EOR) is a well-established prognostic factor for glioma, detailed quantitative EOR data were not available for the prospective validation cohort in this study. Consequently, we could not adjust for EOR in the multivariate analysis for this specific cohort. Future studies with larger sample sizes and complete surgical data are needed to further validate the independent prognostic value of our radiomics model.

In conclusion, we constructed a radiomic prediction model to predict prognosis and chemosensitivity of temozolomide in glioma patients. This prediction models with high stability can be applied to clinical practice.

## Supplementary Information

Supplementary Material 1. Figure S1. Numbers of patients enrolled or excluded in train, validation and prospective group. Numbers of patients enrolled or excluded in train, validation and prospective group and the reason. Figure S2. Comprehensive evaluation of the radiomics prediction model performance across training, validation, and prospective cohorts. The figure is organized into three columns representing the Training Cohort (left), Retrospective Validation Cohort (middle), and Prospective Validation Cohort (right). (A, B) Time-dependent Receiver Operating Characteristic (ROC) curves and Area Under the Curve (AUC) values for predicting 1-year (A) and 3-year (B) overall survival. (C, D) Calibration plots for 1-year (C) and 3-year (D) overall survival. The x-axis represents the predicted survival probability, and the y-axis represents the observed survival probability. (E, F) Decision Curve Analysis (DCA) for 1-year (E) and 3-year (F) overall survival. The x-axis indicates the threshold probability for treatment decision, and the y-axis indicates the net benefit. The red line represents the radiomics model, the gray line represents the assumption that all patients receive treatment, and the horizontal black line represents the assumption that no patients receive treatment. Figure S3. RFs related BPs of tumor and tumor related BPs prediction model. The radar chart showed the counts of BPs most correlated with RFs in apoptosis (A), migration (B), angiogenesis (C) and proliferation process (D). Figure S4. Intratumoral heterogeneity of RFs-associated BPs and verification of prediction accuracy of BPs predictive models by single cell sequencing. (A, B, C) RFs-associated BPs-based t-SNE analysis showed each group of cells was derived from different site of samples. Cells derived from the same site were labeled as same color. No site -related clustering features was observed.

## Data Availability

The data might be made available upon request, and some restrictions will apply.

## References

[1] Jiang T, Mao Y, Ma W, Mao Q, You Y, Yang X, et al. CGCG clinical practice guidelines for the management of adult diffuse gliomas. Cancer Lett. 2016;375(2):263–73. 26966000 10.1016/j.canlet.2016.01.024

[2] Stupp R, Mason WP, van den Bent MJ, Weller M, Fisher B, Taphoorn MJ, et al. Radiotherapy plus concomitant and adjuvant Temozolomide for glioblastoma. N Engl J Med. 2005;352(10):987–96. 15758009 10.1056/NEJMoa043330

[3] Beiko J, Suki D, Hess KR, Fox BD, Cheung V, Cabral M, et al. IDH1 mutant malignant Astrocytomas are more amenable to surgical resection and have a survival benefit associated with maximal surgical resection. Neurooncology. 2014;16(1):81–91. 10.1093/neuonc/not159 PMC3870823 24305719

[4] Hegi ME, Diserens AC, Gorlia T, Hamou MF, de Tribolet N, Weller M, et al. MGMT gene Silencing and benefit from Temozolomide in glioblastoma. N Engl J Med. 2005;352(10):997–1003. 15758010 10.1056/NEJMoa043331

[5] Lu H, Arshad M, Thornton A, Avesani G, Cunnea P, Curry E, et al. A mathematical-descriptor of tumor-mesoscopic-structure from computed-tomography images annotates prognostic- and molecular-phenotypes of epithelial ovarian cancer. Nat Commun. 2019;10(1):764. 30770825 10.1038/s41467-019-08718-9 PMC6377605

[6] Bera K, Braman N, Gupta A, Velcheti V, Madabhushi A. Predicting cancer outcomes with radiomics and artificial intelligence in radiology. Nat Rev Clin Oncol. 2022;19(2):132–46. 34663898 10.1038/s41571-021-00560-7 PMC9034765

[7] Mayerhoefer ME, Materka A, Langs G, Häggström I, Szczypiński P, Gibbs P, et al. Introduction to radiomics. J Nucl Med. 2020;61(4):488–95. 32060219 10.2967/jnumed.118.222893 PMC9374044

[8] Xia T, Zhao B, Li B, Lei Y, Song Y, Wang Y, et al. MRI-Based radiomics and deep learning in biological characteristics and prognosis of hepatocellular carcinoma: opportunities and challenges. J Magn Reson Imaging. 2024;59(3):767–83. 10.1002/jmri.28982 37647155

[9] Xue C, Zhou Q, Xi H, Zhou J, Radiomics. A review of current applications and possibilities in the assessment of tumor microenvironment. Diagn Interv Imaging. 2023;104(3):113–22. 36283933 10.1016/j.diii.2022.10.008

[10] Alizadeh M, Broomand Lomer N, Azami M, Khalafi M, Shobeiri P, Arab Bafrani M, et al. Radiomics: the new promise for differentiating Progression, Recurrence, Pseudoprogression, and radionecrosis in glioma and glioblastoma multiforme. Cancers (Basel). 2023;15(18):4429. 10.3390/cancers15184429 PMC10526457 37760399

[11] Singh G, Manjila S, Sakla N, True A, Wardeh AH, Beig N, et al. Radiomics and radiogenomics in gliomas: a contemporary update. Br J Cancer. 2021;125(5):641–57. 33958734 10.1038/s41416-021-01387-w PMC8405677

[12] van Griethuysen JJM, Fedorov A, Parmar C, Hosny A, Aucoin N, Narayan V, et al. Computational radiomics system to Decode the radiographic phenotype. Cancer Res. 2017;77(21):e104–7. 29092951 10.1158/0008-5472.CAN-17-0339 PMC5672828

[13] Castro DJ, Maurer J, Hebbard L, Oshima RG. ROCK1 Inhibition promotes the self-renewal of a novel mouse mammary cancer stem cell. Stem Cells. 2013;31(1):12–22. 22961723 10.1002/stem.1224 PMC3967738

[14] Shin J, Seo N, Baek SE, Son NH, Lim JS, Kim NK, et al. MRI radiomics model predicts pathologic complete response of rectal cancer following chemoradiotherapy. Radiology. 2022;303(2):351–8. 35133200 10.1148/radiol.211986

[15] Zhang LL, Huang MY, Li Y, Liang JH, Gao TS, Deng B, et al. Pretreatment MRI radiomics analysis allows for reliable prediction of local recurrence in non-metastatic T4 nasopharyngeal carcinoma. EBioMedicine. 2019;42:270–80. 30928358 10.1016/j.ebiom.2019.03.050 PMC6491646

[16] Li Y, Qian Z, Xu K, Wang K, Fan X, Li S, et al. MRI features predict p53 status in lower-grade gliomas via a machine-learning approach. Neuroimage Clin. 2018;17:306–11. 10.1016/j.nicl.2017.10.030 PMC5842645 29527478

[17] Gupta A, Young RJ, Shah AD, Schweitzer AD, Graber JJ, Shi W, et al. Pretreatment dynamic susceptibility contrast MRI perfusion in glioblastoma: prediction of EGFR gene amplification. Clin Neuroradiol. 2015;25(2):143–50. 24474262 10.1007/s00062-014-0289-3 PMC4712066

[18] Qian Z, Li Y, Fan X, Zhang C, Wang Y, Jiang T, et al. Molecular and clinical characterization of IDH associated immune signature in lower-grade gliomas. Oncoimmunology. 2018;7(6):e1434466. 29872572 10.1080/2162402X.2018.1434466 PMC5980422

[19] Li Y, Liu X, Qian Z, Sun Z, Xu K, Wang K, et al. Genotype prediction of ATRX mutation in lower-grade gliomas using an MRI radiomics signature. Eur Radiol. 2018;28(7):2960–8. 29404769 10.1007/s00330-017-5267-0

[20] Liu X, Li Y, Li S, Fan X, Sun Z, Yang Z, et al. IDH mutation-specific radiomic signature in lower-grade gliomas. Aging. 2019;11(2):673–96. 30696801 10.18632/aging.101769 PMC6366985

[21] Liu X, Li Y, Qian Z, Sun Z, Xu K, Wang K, et al. A radiomic signature as a non-invasive predictor of progression-free survival in patients with lower-grade gliomas. Neuroimage Clin. 2018;20:1070–7. 30366279 10.1016/j.nicl.2018.10.014 PMC6202688

[22] Zhou H, Vallieres M, Bai HX, Su C, Tang H, Oldridge D, et al. MRI features predict survival and molecular markers in diffuse lower-grade gliomas. Neurooncology. 2017;19(6):862–70. 10.1093/neuonc/now256 PMC5464433 28339588

[23] Li G, Li L, Li Y, Qian Z, Wu F, He Y, et al. An MRI radiomics approach to predict survival and tumour-infiltrating macrophages in gliomas. Brain. 2022;145(3):1151–61. 35136934 10.1093/brain/awab340 PMC9050568

[24] Sun R, Limkin EJ, Vakalopoulou M, Dercle L, Champiat S, Han SR, et al. A radiomics approach to assess tumour-infiltrating CD8 cells and response to anti-PD-1 or anti-PD-L1 immunotherapy: an imaging biomarker, retrospective multicohort study. Lancet Oncol. 2018;19(9):1180–91. 30120041 10.1016/S1470-2045(18)30413-3

[25] Grossmann P, Stringfield O, El-Hachem N, Bui MM, Rios Velazquez E, Parmar C, et al. Defining the biological basis of radiomic phenotypes in lung cancer. Elife. 2017;6:e23421. 10.7554/eLife.23421 PMC5590809 28731408

[26] De Feyter HM, Behar KL, Corbin ZA, Fulbright RK, Brown PB, McIntyre S, et al. Deuterium metabolic imaging (DMI) for MRI-based 3D mapping of metabolism in vivo. Sci Adv. 2018;4(8):eaat7314. 30140744 10.1126/sciadv.aat7314 PMC6105304

[27] Zhang XN, Yang KD, Chen C, He ZC, Wang QH, Feng H, et al. Pericytes augment glioblastoma cell resistance to Temozolomide through CCL5-CCR5 paracrine signaling. Cell Res. 2021;31(10):1072–87. 34239070 10.1038/s41422-021-00528-3 PMC8486800

[28] Lam MS, Aw JJ, Tan D, Vijayakumar R, Lim HYG, Yada S, et al. Unveiling the influence of tumor microenvironment and Spatial heterogeneity on Temozolomide resistance in glioblastoma using an advanced human in vitro model of the Blood-Brain barrier and glioblastoma. Small. 2023;19(52):e2302280. 37649234 10.1002/smll.202302280

