# Supplementary figures and images for: Radiomics of T2-weighted MRI for pretreatment prediction of prognosis and temozolomide chemosensitivity in glioma
Source: BMC Cancer. 2026 Feb 17;26:398. doi: 10.1186/s12885-026-15726-8 (PMC13019749; doi:10.1186/s12885-026-15726-8)

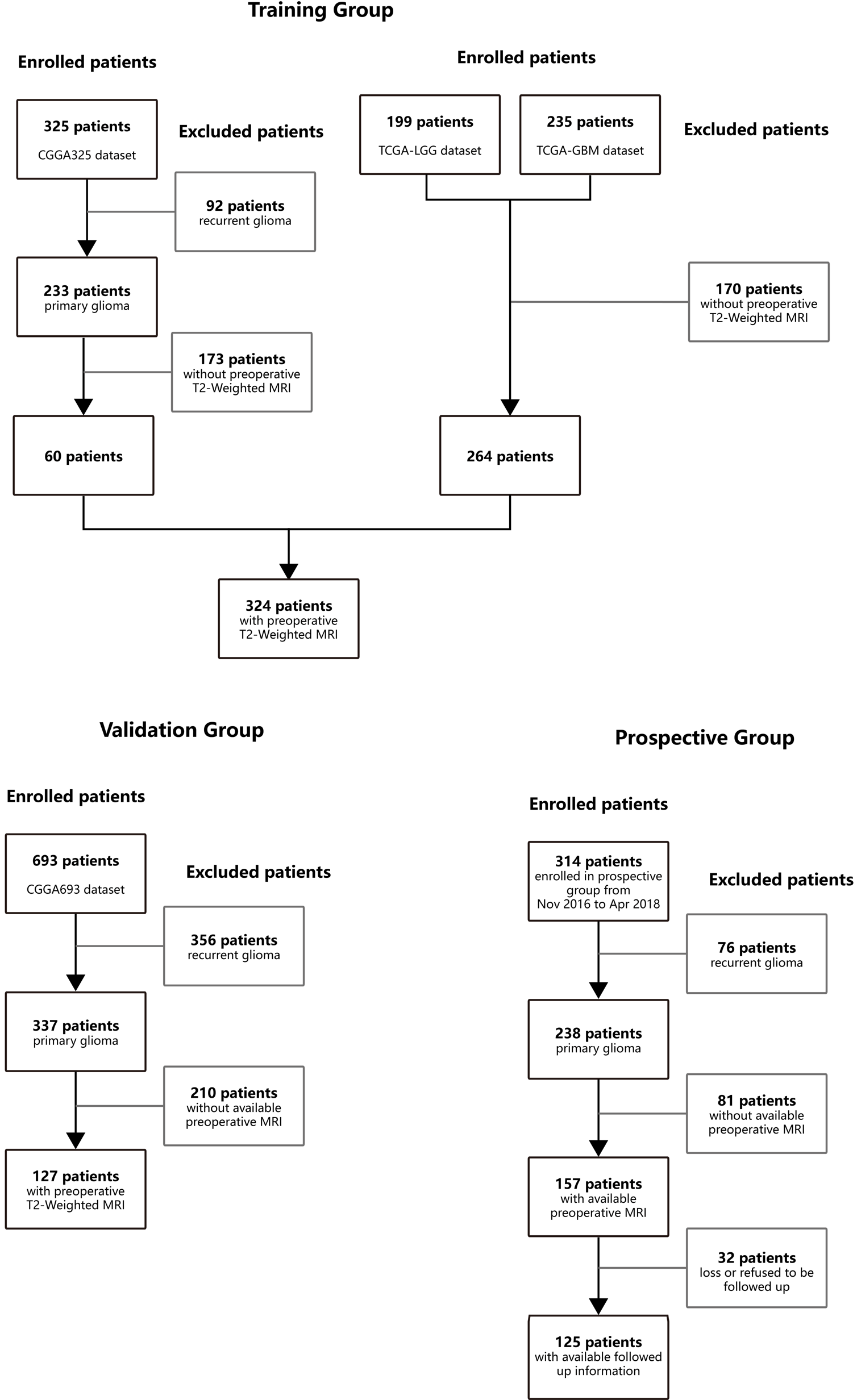

Supplement: Supplementary file 1 — Supplementary Material 1. Figure S1. Numbers of patients enrolled or excluded in train, validation and prospective group. Numbers of patients enrolled or excluded in train, validation and prospective group and the reason. Figure S2. Comprehensive evaluation of the radiomics prediction model performance across training, validation, and prospective cohorts. The figure is organized into three columns representing the Training Cohort (left), Retrospective Validation Cohort (middle), and Prospective Validation Cohort (right). (A, B) Time-dependent Receiver Operating Characteristic (ROC) curves and Area Under the Curve (AUC) values for predicting 1-year (A) and 3-year (B) overall survival. (C, D) Calibration plots for 1-year (C) and 3-year (D) overall survival. The x-axis represents the predicted survival probability, and the y-axis represents the observed survival probability. (E, F) Decision Curve Analysis (DCA) for 1-year (E) and 3-year (F) overall survival. The x-axis indicates the threshold probability for treatment decision, and the y-axis indicates the net benefit. The red line represents the radiomics model, the gray line represents the assumption that all patients receive treatment, and the horizontal black line represents the assumption that no patients receive treatment. Figure S3. RFs related BPs of tumor and tumor related BPs prediction model. The radar chart showed the counts of BPs most correlated with RFs in apoptosis (A), migration (B), angiogenesis (C) and proliferation process (D). Figure S4. Intratumoral heterogeneity of RFs-associated BPs and verification of prediction accuracy of BPs predictive models by single cell sequencing. (A, B, C) RFs-associated BPs-based t-SNE analysis showed each group of cells was derived from different site of samples. Cells derived from the same site were labeled as same color. No site -related clustering features was observed. [file 12885_2026_15726_MOESM1_ESM.zip › supplement material/fig.S1.tif]

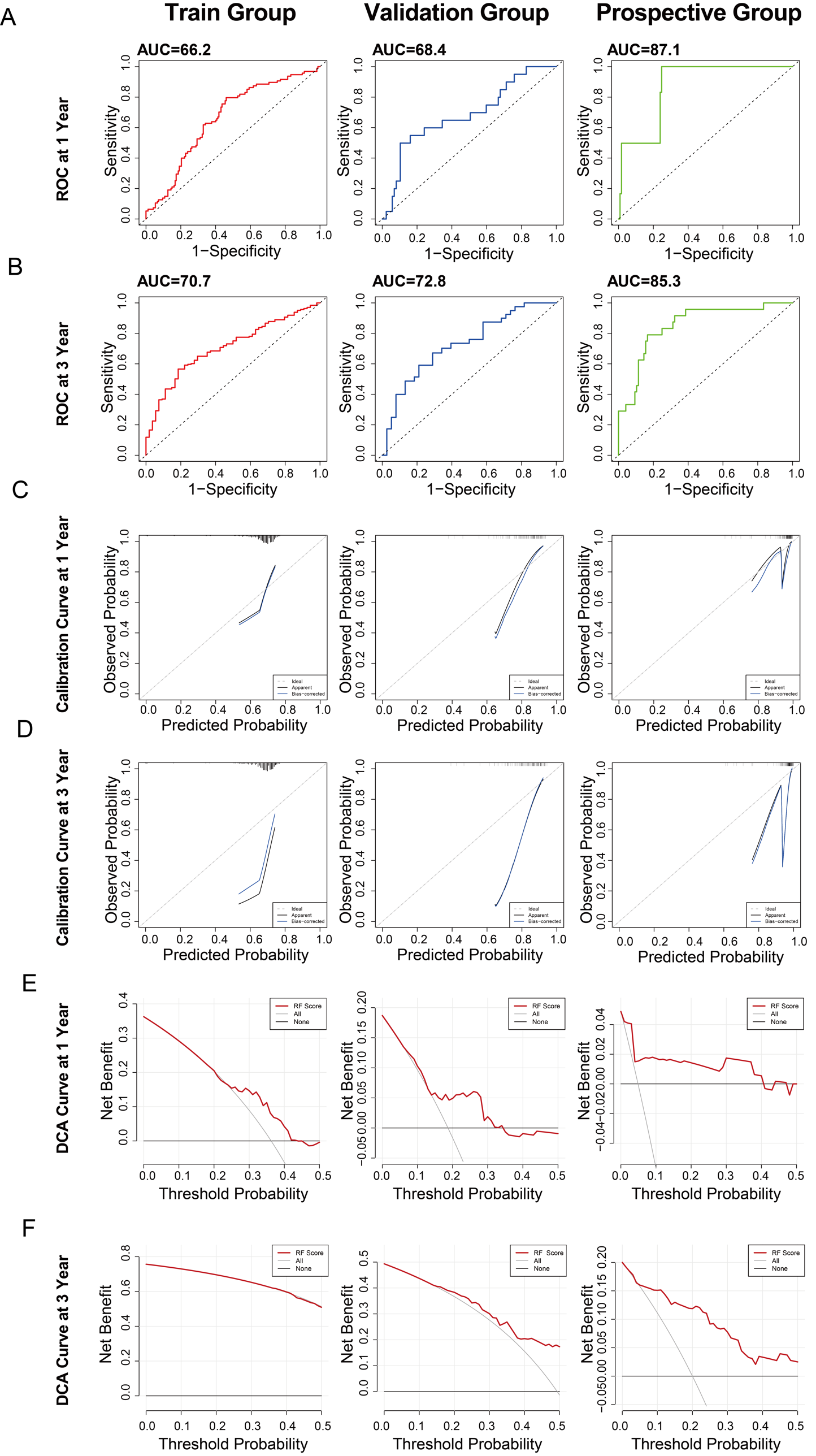

Supplement: Supplementary file 1 — Supplementary Material 1. Figure S1. Numbers of patients enrolled or excluded in train, validation and prospective group. Numbers of patients enrolled or excluded in train, validation and prospective group and the reason. Figure S2. Comprehensive evaluation of the radiomics prediction model performance across training, validation, and prospective cohorts. The figure is organized into three columns representing the Training Cohort (left), Retrospective Validation Cohort (middle), and Prospective Validation Cohort (right). (A, B) Time-dependent Receiver Operating Characteristic (ROC) curves and Area Under the Curve (AUC) values for predicting 1-year (A) and 3-year (B) overall survival. (C, D) Calibration plots for 1-year (C) and 3-year (D) overall survival. The x-axis represents the predicted survival probability, and the y-axis represents the observed survival probability. (E, F) Decision Curve Analysis (DCA) for 1-year (E) and 3-year (F) overall survival. The x-axis indicates the threshold probability for treatment decision, and the y-axis indicates the net benefit. The red line represents the radiomics model, the gray line represents the assumption that all patients receive treatment, and the horizontal black line represents the assumption that no patients receive treatment. Figure S3. RFs related BPs of tumor and tumor related BPs prediction model. The radar chart showed the counts of BPs most correlated with RFs in apoptosis (A), migration (B), angiogenesis (C) and proliferation process (D). Figure S4. Intratumoral heterogeneity of RFs-associated BPs and verification of prediction accuracy of BPs predictive models by single cell sequencing. (A, B, C) RFs-associated BPs-based t-SNE analysis showed each group of cells was derived from different site of samples. Cells derived from the same site were labeled as same color. No site -related clustering features was observed. [file 12885_2026_15726_MOESM1_ESM.zip › supplement material/fig.S2.tif]

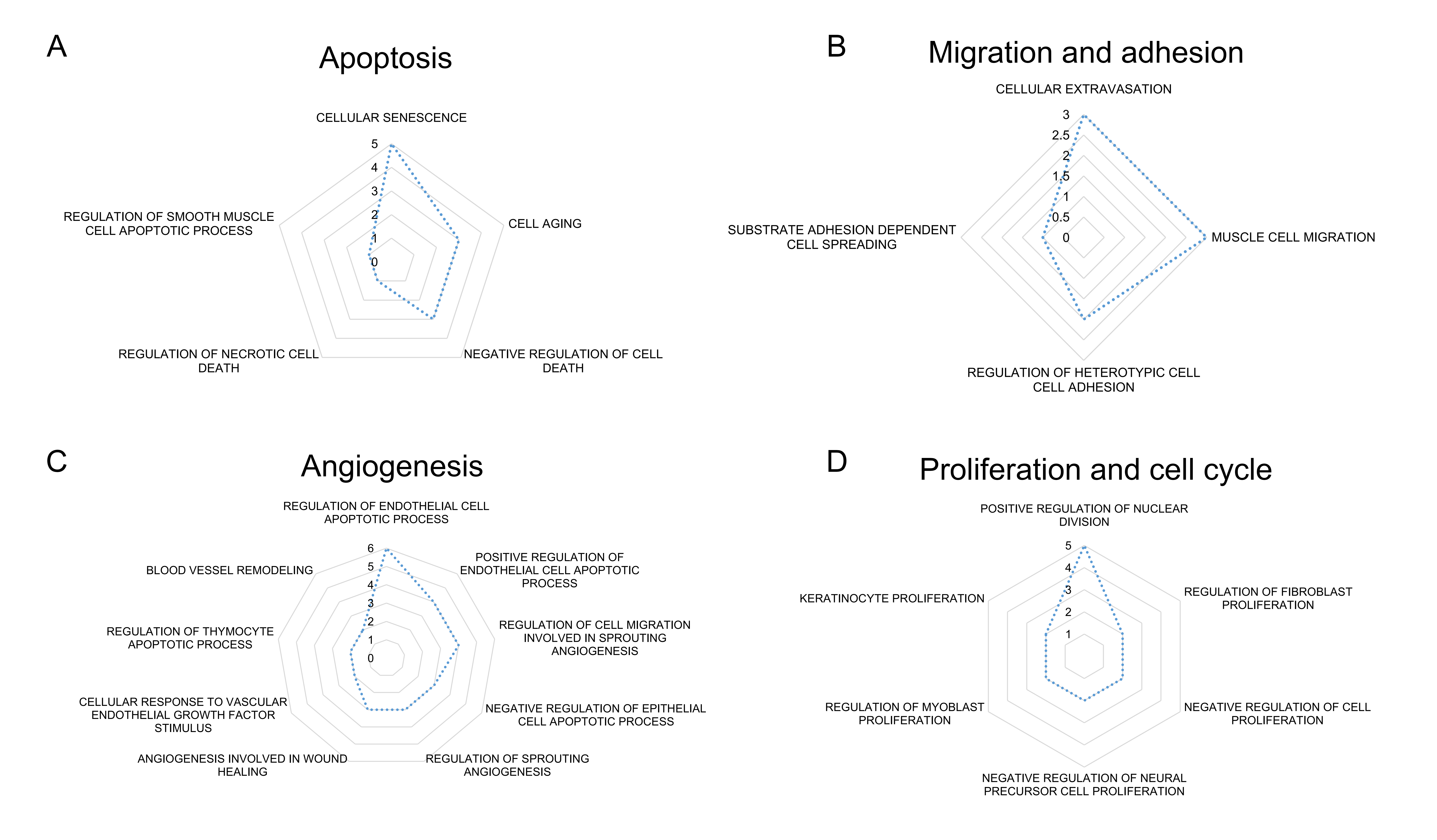

Supplement: Supplementary file 1 — Supplementary Material 1. Figure S1. Numbers of patients enrolled or excluded in train, validation and prospective group. Numbers of patients enrolled or excluded in train, validation and prospective group and the reason. Figure S2. Comprehensive evaluation of the radiomics prediction model performance across training, validation, and prospective cohorts. The figure is organized into three columns representing the Training Cohort (left), Retrospective Validation Cohort (middle), and Prospective Validation Cohort (right). (A, B) Time-dependent Receiver Operating Characteristic (ROC) curves and Area Under the Curve (AUC) values for predicting 1-year (A) and 3-year (B) overall survival. (C, D) Calibration plots for 1-year (C) and 3-year (D) overall survival. The x-axis represents the predicted survival probability, and the y-axis represents the observed survival probability. (E, F) Decision Curve Analysis (DCA) for 1-year (E) and 3-year (F) overall survival. The x-axis indicates the threshold probability for treatment decision, and the y-axis indicates the net benefit. The red line represents the radiomics model, the gray line represents the assumption that all patients receive treatment, and the horizontal black line represents the assumption that no patients receive treatment. Figure S3. RFs related BPs of tumor and tumor related BPs prediction model. The radar chart showed the counts of BPs most correlated with RFs in apoptosis (A), migration (B), angiogenesis (C) and proliferation process (D). Figure S4. Intratumoral heterogeneity of RFs-associated BPs and verification of prediction accuracy of BPs predictive models by single cell sequencing. (A, B, C) RFs-associated BPs-based t-SNE analysis showed each group of cells was derived from different site of samples. Cells derived from the same site were labeled as same color. No site -related clustering features was observed. [file 12885_2026_15726_MOESM1_ESM.zip › supplement material/fig.S3.tif]

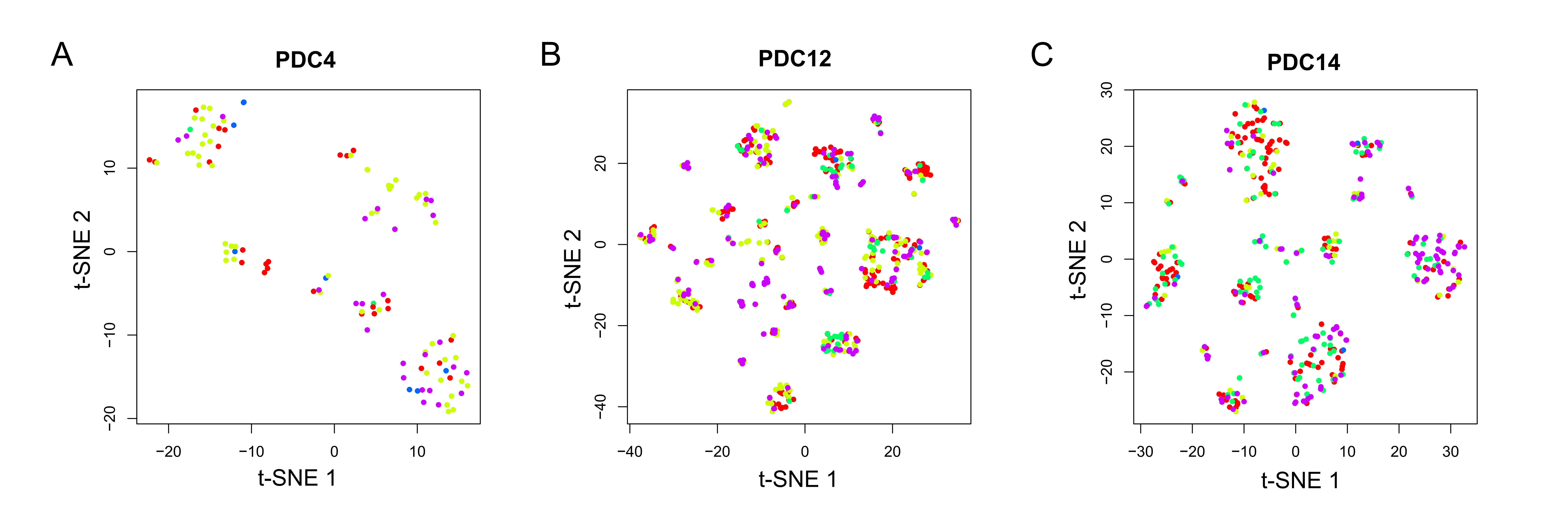

Supplement: Supplementary file 1 — Supplementary Material 1. Figure S1. Numbers of patients enrolled or excluded in train, validation and prospective group. Numbers of patients enrolled or excluded in train, validation and prospective group and the reason. Figure S2. Comprehensive evaluation of the radiomics prediction model performance across training, validation, and prospective cohorts. The figure is organized into three columns representing the Training Cohort (left), Retrospective Validation Cohort (middle), and Prospective Validation Cohort (right). (A, B) Time-dependent Receiver Operating Characteristic (ROC) curves and Area Under the Curve (AUC) values for predicting 1-year (A) and 3-year (B) overall survival. (C, D) Calibration plots for 1-year (C) and 3-year (D) overall survival. The x-axis represents the predicted survival probability, and the y-axis represents the observed survival probability. (E, F) Decision Curve Analysis (DCA) for 1-year (E) and 3-year (F) overall survival. The x-axis indicates the threshold probability for treatment decision, and the y-axis indicates the net benefit. The red line represents the radiomics model, the gray line represents the assumption that all patients receive treatment, and the horizontal black line represents the assumption that no patients receive treatment. Figure S3. RFs related BPs of tumor and tumor related BPs prediction model. The radar chart showed the counts of BPs most correlated with RFs in apoptosis (A), migration (B), angiogenesis (C) and proliferation process (D). Figure S4. Intratumoral heterogeneity of RFs-associated BPs and verification of prediction accuracy of BPs predictive models by single cell sequencing. (A, B, C) RFs-associated BPs-based t-SNE analysis showed each group of cells was derived from different site of samples. Cells derived from the same site were labeled as same color. No site -related clustering features was observed. [file 12885_2026_15726_MOESM1_ESM.zip › supplement material/fig.S4.tif]
